# A Hymn to Neurosurgery and Neuroscience

**DOI:** 10.1371/journal.pbio.0030214

**Published:** 2005-06-14

**Authors:** Richard Smith

## Abstract

Ian McEwan brings insights into neurosurgery and mind/brain duality to his latest book, *Saturday.*

**Figure pbio-0030214-g001:**
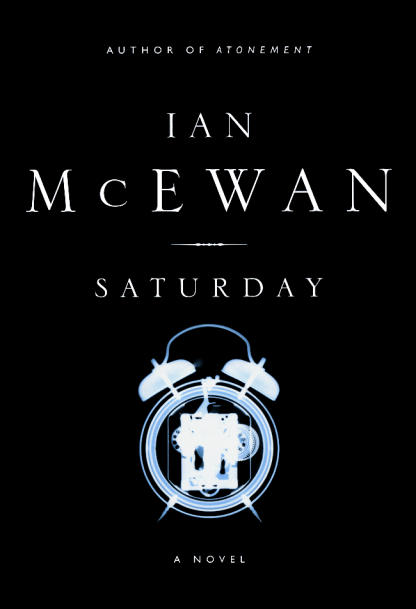


“Marketeers prove…every scientific term you use represents two thousand readers putting down the magazine and turning on a rerun of *I Love Lucy*,” says a world-weary editor in David Mitchell's Russian doll of a novel *Cloud Atlas*. The marketeers of Ian McEwan (if he has any) clearly don't agree. His latest novel, *Saturday*, is saturated with medical and scientific terms, many of them unexplained. The book is, indeed, a celebration of neurosurgery and neuroscience with almost all the climactic moments in the book hinging around injury to the brain. And reaching beyond neuroscience, McEwan continues the 19th century debate between T. H. Huxley and the Bishop of Oxford over science versus faith, placing himself firmly on the side of Huxley.[Fig pbio-0030214-g001]

McEwan is widely considered to be Britain's pre-eminent novelist. His last book, *Atonement*, moved him ahead of the pack, and *Saturday* puts him further out front. Following the model of James Joyce's *Ulysses*, the book tells the story of one day in the life of a man—only without the stylistic fireworks and complexity of Joyce's book. McEwan's character is not a drunkard, although he loves good wine and good food, but a neurosurgeon, Henry Perowne, and his day is full of incident and drama (which I won't reveal). But nobody should fear that the book is a lecture on neurosurgery and neuroscience. It tells a compelling story and says much that is insightful about human relationships—and at the crucial moment it is poetry not science that saves the heroes. Indeed, I read the book before I was asked to review it, and it was only as I read it for the second time that I grasped that every few pages there were scientific references. The science underpins rather than undermines the story. Wisdom is imparted lightly.

The brain is a worthy subject for a writer at the height of his powers. In the acknowledgments, McEwan thanks several doctors and scientists for their support and mentions spending two years watching neurosurgeon Neil Kitchen operate. David Lodge, another British writer, tackled consciousness in his book *Thinks* but produced a novel that did feel too much like a lecture on psychology, albeit an entertaining and understandable one. McEwan emphasises that the mechanisms of the brain—which like an expensive car is intricate but “mass produced nevertheless, with more than six billion in circulation”—are still largely unknown. We don't know “how it holds experiences, memories, dreams, and intentions”, but Perowne is sure that the brain's fundamental secret will be laid open one day. The result will not be man exposed as a machine but rather that such understanding will be magnificent and uplifting. “The actual, not the magical, should be the challenge.” This is the cry of science, particularly when the actual is so magical, and surely need not be incompatible with faith.

The followers of the Bishop of Oxford take, nonetheless, something of a beating. Perowne, clearly a hero to McEwan (and perhaps too much so), believes that “the primitive thinking of the supernaturally inclined amounts to what his psychiatric colleagues call a problem…an inability to contemplate your own unimportance.” People resort to the supernatural through “insufficient imagination”. It's too easy and comfortable to believe that “an all-knowing supernatural force had allotted people to their stations in life”. Indeed, it's “a form of anosognosia, a useful psychiatric term for a lack of awareness of one's own condition”. To insult your opponent is rarely the best policy—but can be hard to resist.

Perowne is inevitably for McEwan a materialist and even a determinist. “There is much in human affairs that can be accounted for at the level of the complex molecule.” For one of the main characters his “misfortune lies within a single gene, in an excessive repeat of a single sequence—CAG. Here's biological determinism in its purest form.” (Readers clever enough to know the result of that sequence repeated excessively will know the condition of one character, but many other conditions are described in equal detail. Indeed, a whole operating list is vividly described close to the beginning of the book.) McEwan is scornful of those who would cure genetic diseases. “*It is written*,” he writes with the words in italic and in parody of religious texts. “No amount of love, drugs, Bible classes, or prison sentencing can cure [the character with the genetic disease].”

Despite the attention to scientific detail, *Saturday* provides more insights into neurosurgery and neurosurgeons than it does into science. McEwan is fascinated that neurosurgery is essentially plumbing when its object is brilliant circuitry. Who would want a plumber working on their computer? Yet often it turns out well. Perowne does about 300 cases a year. “Some fail, a handful endure with their lights a little fogged, but most thrive, and many return to work in some form; work—the ultimate badge of health.” I found myself wondering if the success rates are so good. I don't think that they are. My main memory of neurosurgery was a professor angrily pulling bits of skull from the brain of a patient with a severe head injury. The result was not good. McEwan attributes a different view of neurosurgery to psychiatrists: “The neurosurgeons are blundering arrogant fools with blunt instruments, bone-setters set loose on the most complex object in the known universe.” (Mitchell in *Cloud Atlas*, which is more cynical but also funnier, says this: “To us [surgeons] people aren't sacred beings crafted in the Almighty's image, no, people are joints of meat; diseased, leathery meat, yes, but meat ready for the skewer and the spit.”)

For McEwan his neurosurgeon is something close to a genius. Perowne sees himself as artless and is amazed that he has fathered a published poet and one of England's most promising blues musicians. How did this happen? McEwan has Perowne express his idea of genius: “Work that you cannot begin to imagine achieving yourself, that displays a ruthless, nearly inhuman element of self-enclosed perfection.” Perowne is thinking of Bach, Beethoven, Mozart, Schubert, Gil Evans, Miles Davis, John Coltrane, Cezanne, and Einstein, but to the reader Perowne fits his own definition of genius. This is not so odd: a friend of mine, Charlie Wilson, a neurosurgeon with particular skills in pituitary surgery, was described a few years ago by a magazine as a physical genius. He was compared with Yo Yo Ma. Perowne likes to operate to Bach—to the Goldberg Variations played not by Glenn Gould (showy and unorthodox) but by Angela Hewitt (wise and silky)—and McEwan interweaves descriptions of a difficult operation and the music. We observe Bach, Hewitt, and Perowne all working together, all geniuses.

Whether or not they are geniuses, neurosurgeons—like all surgeons—love to operate. The rest of life—and particularly the outpatient clinic—is an anticlimax. I remember as a junior surgeon (one who was so junior that I did only one—unfortunately disastrous—operation alone) calling in the senior surgeon in the middle of the night to operate on a man with a burst abdominal aneurysm. We operated until dawn, and I never saw that saturnine surgeon so happy—although he didn't thank me for pointing it out. For Perowne, called in by his own junior in the middle of a dramatic night, “operating never wearies him—once busy…he experiences a superhuman capacity, more like a craving, for work.” The operating theatre is “home from home. Though sometimes things go wrong, he can control outcomes here, he has resources, controlled conditions.” His possible genius and his contentment in operating are in contrast to the mess and uncertainty of his non-surgical life, of all our lives.

But if neurosurgery is close to genius it is also close to sex. Perowne met his wife when she was admitted with “pituitary apoplexy” (which is well explained in the book), and the text moves quickly from the intimacy of being inside her skull to sexual intimacy (with no suggestion of misconduct). After he finishes a difficult operation, which he did after being awake for nearly 24 hours and having had many adventures, “even his awareness of his own existence has vanished. He's been delivered into a pure present, free of the weight of the past or anxieties about the future…It's a little like sex, in that he feels himself in another medium….”

This wonderful book might dramatically increase recruitment into neurosurgery.

